# Molecular Insights into Macromolecules Structure, Function, and Regulation

**DOI:** 10.3390/ijms25105296

**Published:** 2024-05-13

**Authors:** Zhiwei Yang, Jiasheng Zhao

**Affiliations:** MOE Key Laboratory for Nonequilibrium Synthesis and Modulation of Condensed Matter, School of Physics, Xi’an Jiaotong University, Xi’an 710049, China; zjs3123333001@stu.xjtu.edu.cn

Macromolecules exhibit ordered structures and complex functions in an aqueous environment with strong thermodynamic fluctuations. The all-atom spatial–temporal structures and dynamics characteristics of macromolecules have become the key to understanding the internal relationship between the structure and function, molecular mechanisms, and functional regulation of related systems [1]. On this point, the intention of this Special Issue is to discuss the structural prediction/modeling and biological function of macromolecules with a variety of computational approaches and experimental strategies in order to study their molecular mechanisms and mutual regulatory interactions (e.g., with nucleic acids, proteins, and relevant condensed matter) [2,3,4].

Under normal circumstances, macromolecules are functionally complex with considerable structural dynamics, and their full-length structures have considerable potential to reduce some of the uncertainty in their intrinsically dynamic behavior [5]. Maria J. Solares and Deborah F. Kelly determined the full-length structures of both monomeric and dimeric forms of p53, a tumor suppressor protein, by using the dynamics simulations/flexible fitting approaches with electron density maps [6]. The complete structural models contribute to the inquiry of structural alterations caused by seven common mutations of p53, which result in aberrant conformations of the DNA-binding domain or impairments in proper DNA interactions, ultimately leading to cellular carcinogenesis. Another manuscript offered some viewpoints to the allosteric mechanisms of the lung-enriched p53 mutants V157F and R158L throughout the all-atom explicit solvent molecular dynamics (MD) simulations and the completely ordered region of the DNA-binding domain [7]. Through these two studies, we can gain molecular-level insights into the conformational mechanisms of p53 mutants to help the redesign of structure-based targeted therapies. The research also indicated that MD simulation with the constraint of experimental structural parameters is a powerful tool for understanding and predicting the dynamic behavior of macromolecules at the atomic level, and can provide more accurate and reliable insights into the properties and functions of macromolecules, particularly those with complex and dynamic microstructures (Figure 1) [8].

By delving into the intrinsically dynamic behavior of macromolecules, we can pinpoint the pivotal nodes of dynamic evolution to uncover the physical properties and regularities of the structure–function relationship to precisely identify the active sites on the binding interfaces of macromolecules, which are crucial for ligand binding and allosteric regulation (Figure 1). By leveraging this knowledge, we can strategically design and rigorously validate drugs that target these potential binding regulatory regions (binding pockets). As is known, the spike protein of severe acute respiratory syndrome coronavirus-2 (SARS-CoV-2) mediates viral entry into the host cells, consequently emerging as an important target of vaccine/drug development to prevent and treat the disease. In Yizhen Zhao et al. 2023 [9], the in situ full-length structures of the spike trimer in both closed (inactive) and open (active) states were constructed with the constraints of electron density maps, and then various regions with the structural difference and dynamism were identified as the potential binding regulatory regions (binding pockets) that could enhance the propensity of proteins to adopt an inactive conformation. Targeting the newly defined binding pocket, five ligands with potential bioactivities were selected with the combination of docking and MD simulations, based on the conformational changes upon ligand binding. A similar research approach was also reflected in the structural dynamic studies of the NS2B/NS3 protease of Kyasanur Forest Disease virus (KFDV), which leads to the onset of severe hemorrhagic fever in humans [10]. As for the study of cholesteryl ester transfer protein (CETP), which is a promising target for mitigating cardiovascular diseases (CVD), the absence of dynamic structural characteristics under physiological conditions (authentic CETP) impedes a comprehensive understanding of the lipid transfer mechanisms and hampers the development of CETP pharmacologic inhibition. To address this, the structural differences between authentic CETP and mutant CETP (a fully active mutation used for crystal structure elucidation) were studied with the MD simulations and electron microscope (EM) data [11]. The results revealed that the authentic CETP is more flexible and conducive to the formation of a continuous tunnel in the “neck” region, providing a more favorable lipid exchange environment in contrast to the mutant CETP, thereby resulting in a greater risk of CVD. Additionally, virtual screening and MD simulation approaches were employed targeting the N- and C-terminus of CETP to discover the potential CETP inhibitor that disrupts the hydrophobic continuous tunnel.

Concurrently, other methods for structural determination, such as X-ray crystallography, nuclear magnetic resonance (NMR), and infrared broad spectrum, along with biochemical assays, also provide a robust and complementary boost to the characterization and understanding of macromolecular dynamics and interactions [12,13]. Si-Bo Chen et al. 2023 [12] revealed that a novel bacterial dipeptidyl peptidase III (*Co*DPP III) exists in a dimeric and closed conformation in the absence of a ligand and accumulates peptides and amino acids to serve as an A-signal, as evidenced by biochemical characterization and crystallization. Konstantin O. Muranov et al. 2023 [13] experimentally determined that α-crystallin exhibits higher molecular chaperone activity than αH-crystallin. Moreover, the refolding of αH-crystallin corrects misfolding and enhances its molecular chaperone activity, offering meaningful prospects for drug development for cataracts.

As we delve deeper into the molecular underpinnings of macromolecules, the interplay between structure, function, and regulation becomes increasingly apparent. This evolution has driven the development of drug discovery from the conventional static/steady-structure paradigm to a dynamic conformation-centric approach, propelled by the synergistic advancements in molecular simulations and artificial intelligence (AI) methods [14], which are reshaping our understanding and manipulation of macromolecules at an unprecedented scale. We sincerely hope that this work will contribute to the study of macromolecular interactions, as well as the evolution, adaptation, and change of living systems.

## Figures and Tables

**Figure 1:**
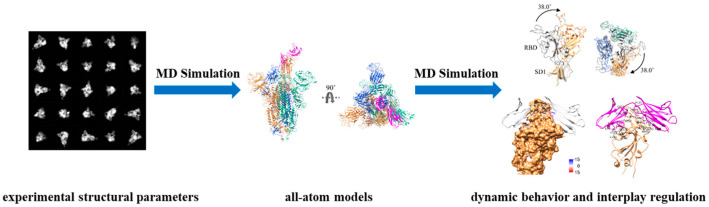
Schematic overview of molecular dynamics (MD) simulation with experimental structural constraints to realize the dynamic behavior and regulation of macromolecules.

## References

[1] Laage D., Elsaesser T., Hynes J.T. (2017). Water Dynamics in the Hydration Shells of Biomolecules. Chem. Rev..

[2] Lee G., Iwase T., Matsumoto S., Nabil A., Ebara M. (2023). Development of Apoptotic-Cell-Inspired Antibody–Drug Conjugate for Effective Immune Modulation. Int. J. Mol. Sci..

[3] Qian Z., Shi D., Zhang H., Li Z., Huang L., Yan X., Lin S. (2024). Transcription Factors and Their Regulatory Roles in the Male Gametophyte Development of Flowering Plants. Int. J. Mol. Sci..

[4] Wang Y., Wang H., Zhang S., Yang Z., Shi X., Zhang L. (2024). Exploration of the Character Representation of DNA Chiral Conformations and Deformations via a Curved Surface Discrete Frenet Frame. Int. J. Mol. Sci..

[5] Seitz E., Acosta-Reyes F., Maji S., Schwander P., Frank J. (2021). Geometric machine learning informed by ground truth: Recovery of conformational continuum from single-particle cryo-EM data of biomolecules. bioRxiv.

[6] Solares M.J., Kelly D.F. (2022). Complete Models of p53 Better Inform the Impact of Hotspot Mutations. Int. J. Mol. Sci..

[7] Lei J., Li X., Cai M., Guo T., Lin D., Deng X., Li Y. (2022). Insights into Allosteric Mechanisms of the Lung-Enriched p53 Mutants V157F and R158L. Int. J. Mol. Sci..

[8] Schlick T., Portillo-Ledesma S., Myers C.G., Beljak L., Chen J., Dakhel S., Darling D., Ghosh S., Hall J., Jan M., Dill K.A. (2021). Biomolecular Modeling and Simulation: A Prospering Multidisciplinary Field. Annual Review of Biophysics, Vol 50, 2021.

[9] Zhao Y., Zhao Y., Xie L., Li Q., Zhang Y., Zang Y., Li X., Zhang L., Yang Z. (2023). Identification of Potential Lead Compounds Targeting Novel Druggable Cavity of SARS-CoV-2 Spike Trimer by Molecular Dynamics Simulations. Int. J. Mol. Sci..

[10] Kandagalla S., Kumbar B., Novak J. (2023). Structural Modifications Introduced by NS2B Cofactor Binding to the NS3 Protease of the Kyasanur Forest Disease Virus. Int. J. Mol. Sci..

[11] Zhao Y., Hao D., Zhao Y., Zhang S., Zhang L., Yang Z. (2023). Dissecting the Structural Dynamics of Authentic Cholesteryl Ester Transfer Protein for the Discovery of Potential Lead Compounds: A Theoretical Study. Int. J. Mol. Sci..

[12] Chen S.-B., Zhang H., Chen S., Ye X.-F., Li Z.-K., Liu W.-D., Cui Z.-L., Huang Y. (2023). Structural and Functional Characterization of a New Bacterial Dipeptidyl Peptidase III Involved in Fruiting Body Formation in Myxobacteria. Int. J. Mol. Sci..

[13] Muranov K.O., Poliansky N.B., Borzova V.A., Kleimenov S.Y. (2023). Refolding Increases the Chaperone-like Activity of αH-Crystallin and Reduces Its Hydrodynamic Diameter to That of α-Crystallin. Int. J. Mol. Sci..

[14] Krishna R., Wang J., Ahern W., Sturmfels P., Venkatesh P., Kalvet I., Lee G.R., Morey-Burrows F.S., Anishchenko I., Humphreys I.R. (2024). Generalized biomolecular modeling and design with RoseTTAFold All-Atom. Science.

